# Optimizing PET/CT protocols: is 60-minute [18 F]F-FDG uptake sufficient for cardiac sarcoidosis?

**DOI:** 10.1186/s13550-026-01380-5

**Published:** 2026-01-20

**Authors:** Giulia Metzger, Bettina Heidecker, Jonas Kaufmann, Markus Galler, Christian Bayerl, Hans Jochens, Norman Limberg, Imke Schatka, Thula Cannon Walter-Rittel, Julian Rogasch, Winfried Brenner, Ulf Landmesser, Holger Amthauer, Christian Furth

**Affiliations:** 1 Charité – Universitätsmedizin Berlin, corporate member of Freie Universität Berlin and Humboldt-Universität zu Berlin, Department of Nuclear Medicine, Berlin, Germany; 2 Charité – Universitätsmedizin Berlin, corporate member of Freie Universität Berlin and Humboldt-Universität zu Berlin, Department of Cardiology, Angiology and Intensive Care Medicine, Deutsches Herzzentrum der Charité, Berlin, Germany; 3 Charité – Universitätsmedizin Berlin, corporate member of Freie Universität Berlin and Humboldt-Universität zu Berlin, Department of Radiology, Berlin, Germany; 4 Charité – Universitätsmedizin Berlin, corporate member of Freie Universität Berlin and Humboldt-Universität zu Berlin, Department of Nuclear Medicine, Berlin, Germany

**Keywords:** FDG PET/CT, Cardiac sarcoidosis, Uptake time, Diagnostic accuracy, Reliability

## Abstract

**Background:**

[^18^F]F-FDG PET/CT is an established imaging modality for diagnosing cardiac sarcoidosis (CS). While a 90-minute uptake time is commonly recommended to enhance target-to-background ratio, its added diagnostic value remains unclear. This study aimed to compare the diagnostic performance of 60-minute versus 90-minute uptake times. Eighty-seven patients (45 females, 42 males) with suspected CS underwent whole-body FDG PET/CT at 60 min post-injection (p.i.), followed by an additional chest scan at 90 min p.i. Patient preparation included a low-carbohydrate diet, prolonged fasting, and weight-based heparin administration. Three blinded readers with varying experience independently assessed the scans using binary classification for typical sarcoidosis-related FDG uptake, provided adequate myocardial glucose suppression was achieved. Inter- and intrarater agreement were analyzed using Fleiss’ and Cohen’s κ, respectively. Diagnostic accuracy was determined by majority vote, using Japanese Circulation Society (JCS) criteria as the reference standard.

**Results:**

Interrater agreement was substantial (Fleiss’ κ = 0.690–0.693), and intrarater agreement ranged from substantial to almost perfect (Cohen’s κ = 0.703–0.899). Among patients with sufficient myocardial suppression, diagnostic accuracy was 97% (*n* = 62) at 60 min and 92% (*n* = 65) at 90 min. No statistically significant differences were observed between the two time points (*p* = 0.22).

**Conclusion:**

FDG PET/CT with a 60-minute uptake time offers diagnostic accuracy comparable to that of a 90-minute uptake for CS detection, provided adequate myocardial suppression is achieved. Shorter uptake protocols may streamline workflow and improve patient comfort without compromising diagnostic integrity.

**Supplementary Information:**

The online version contains supplementary material available at 10.1186/s13550-026-01380-5.

## Background

Sarcoidosis is a granulomatous multi-system disorder of unknown etiology resulting in the formation of non-caseating granulomas. Disease manifestations and the clinical course of sarcoidosis are wide-ranging and heterogenous, although a majority of cases affect the respiratory system. Despite likely being underdiagnosed in clinical settings, cardiac sarcoidosis (CS) is the most common cause of mortality in patients with sarcoidosis, which is contributed to sudden cardiac death and congestive heart failure in 40–70% [1–3]. Hence, an early diagnosis of CS even in subclinical patients is paramount to improve clinical outcomes in these patients.

Depending on the location, the activity and the extent of cardiac infiltration, patients can present with high-grade conduction disorders (with predominant involvement of the ventricular septum), sustained or non-sustained ventricular arrhythmia (sVT/nsVT, related to right ventricular infiltration), valve insufficiencies or left ventricular dysfunction, the latter of which is related to myocardial fibrosis [4–6]. Cardiac involvement with sarcoidosis should be considered in younger patients (< 55 years) with cardiac symptoms or in patients with known extracardiac sarcoidosis and newly developed cardiac arrhythmia or heart failure [7].

The diagnosis of CS, especially in cases with isolated CS, remains challenging. To streamline the diagnosis of CS, several clinical diagnostic criteria have been established by the Heart Rhythm Society (HRS), the World Association of Sarcoidosis and Other Granulomatous Diseases (WASOG) and the Japanese Circulation Society (JCS), incorporating patient history, symptoms, clinical findings in electrocardiogram (ECG) and echocardiography, biomarkers and advanced imaging methods like cardiac MRI (cMRI) and FDG PET/CT [8–10]. The granulomatous myocardial infiltration in CS is associated with multifocal FDG accumulation in a so-called ‘patchy pattern’ [11]. To ensure the detection of disease-related FDG-uptake, patient preparation is essential to suppress the physiological myocardial glucose metabolism. Recommendations to suppress physiological myocardial glucose utilization, include a prolonged fasting period of at least 12 h [12], adherence to a strict high-fat, low-carbohydrate diet [13], and, when possible, weight-adjusted intravenous administration of unfractionated heparin (UFH) prior to FDG administration [14]. Regarding FDG uptake time, current guidelines recommend 60 to 90 min, with evidence suggesting that 90 min enhances the target-to-blood pool ratio (TBR) by reducing residual blood pool activity, hereby improving myocardial lesion contrast and diagnostic accuracy [8, 10, 15, 16]. However, efficiency and cost-effectiveness in healthcare are gaining importance. Shorter uptake times could streamline workflows, reduce costs, and improve patient experience. Despite the assumed benefits of 90-minute uptake, robust comparative studies validating its superiority over 60-minute uptake protocols are lacking.

This study systematically compares the diagnostic accuracy of FDG PET/CT at 60 min versus 90 min uptake time for detecting cardiac sarcoidosis, assessing intra- and inter-reader agreement in a blinded multi-reader setting and evaluating the impact of uptake time on diagnostic consistency.

## Materials and methods

### Patient cohort

The study group consisted of 87 patients (female, 45; male, 42) between 25 and 87 (mean, 55) years, who were referred to our department for FDG PET/CT between March 2023 and May 2024 for the assessment of extent and severity of CS. The inclusion criteria comprised clinical findings and patient histories consistent with either a suspected diagnosis of isolated CS (n, 47), cardiac involvement in histologically confirmed extracardiac sarcoidosis (extraCS, n, 20), or confirmed CS under immunosuppressive treatment for follow-up evaluation (FU; n, 20). Exclusion criteria included blood glucose levels exceeding 200 mg/dL, intake of more than 20 mg of prednisone per day and a lack adherence to the recommended dietary measures. This monocentric, retrospective study was performed in line with the principles of the Declaration of Helsinki and has been approved by the local ethics committee (EA4/106/24). Informed consent was obtained from all individual participants included in the study. Aggregated patient data is provided in Table 1.

### Patient preparation

Patient preparation and recommended dietary measures are based on increasing the provision of free fatty acids to induce a metabolic shift in the myocardium, minimizing the physiological myocardial glucose utilization and improving the TBR [15]. A prolonged fasting period of a minimum of 12 h, a low-carbohydrate, high-fat diet for at least 48 h and IV administration of unfractioned heparin (UFH) was requested in accordance with the joint Society of Nuclear Medicine and Molecular Imaging/American Society of Nuclear Cardiology (SNMMI-ASNC) and the European Society of Cardiology (ESC) expert consensus statements [15, 16].

### [^18^F]F-FDG PET/CT imaging

The administered activity of [^18^F]F-FDG ranged from 147 to 417 MBq (mean, 242 MBq). A digital PET device (General Electric^®^ Healthcare, Discovery MI, Chicago, Illinois, United States; silicon photomultipliers [SiPM], 3-ring detector setting, Time of flight [TOF] capability, system sensitivity of 7.3 cps/kBq) was used for PET/CT imaging. Scans were conducted with acquisition times of 2 to 3 min per bed position. Whole body PET acquisition from the base of skull to the proximal femur was obtained after a median of 62 min (range, 59–65 min) post injection (p.i.) of the tracer. The PET/CT scans always included a non-enhanced low-dose CT for attenuation correction. Following whole-body imaging, an additional thoracic acquisition was conducted with a median delay of 91 min p.i. (range, 88–93 min). PET raw data were reconstructed using iterative reconstruction according to the manufacturer´s settings. PET data were corrected for randoms, scatter, attenuation and dead time.

### Image evaluation

For visual interpretation fused imaging data of the thorax (i.e., PET and low-dose CT) 60 and 90 min p.i. were randomized and evaluated by one experienced, board-certified radiologist and nuclear physician (Reader 1, > 15 years of experience), one experienced, board-certified nuclear physician (Reader 2, > 6 years of experience) and one nuclear physician resident (Reader 3, > 2 years of experience), each of whom were blinded to uptake time, patient history and medication. If a pacemaker or other cardiac devices were present, the readers were provided with the non-attenuation corrected (NAC) images to rule out artifacts arising from attenuation overcorrection.

Image interpretation followed a structured two-step approach. In a first step, the readers evaluated whether myocardial glucose metabolism was adequately suppressed. Adequate suppression was defined as complete or near-complete absence of diffuse physiological FDG uptake in the left ventricular myocardium, while minimal residual uptake confined to the papillary muscles or the basal septum was still considered acceptable. Only scans that fulfilled this criterion according to the respective reader were subjected to further analysis. Scans that were not classified as adequately suppressed were excluded from further evaluation, as residual physiological uptake could not be reliably distinguished from pathological myocardial inflammation. These scans were therefore considered as drop-outs. The readers received prior training to recognize insufficient suppression patterns.

In a second step, the readers assessed whether the images demonstrated a sarcoidosis-typical “patchy” pattern. This was characterized as heterogeneous, focal or focal-on-diffuse FDG uptake with marked regional differences in intensity, usually localized to the basal and mid-ventricular segments, with a predilection for septal, lateral, and anterior wall involvement [17]. The uptake had to be clearly above background blood pool activity and not attributable to attenuation artifacts. Diffuse uptake patterns were not regarded as indicative of cardiac sarcoidosis. Per reader, a binary assessment regarding the presence or absence of sarcoidosis-typical tracer distribution was performed. Intra- and inter-reader agreement was assessed. Intra-reader agreement referred to the consistency of image interpretation between the 60- and 90-minute scans for each individual reader, whereas inter-reader agreement reflected the concordance among the three readers at each respective time point.

In addition, a majority-vote approach was applied to establish a consensus-based reference standard. This approach has been employed in prior imaging studies of cardiac sarcoidosis [18]. By reducing the impact of individual reader bias, majority voting provides a more consistent basis for scan classification.

This consensus outcome could then be compared with the reference diagnosis defined by our defined gold standard (described below) in order to calculate diagnostic test characteristics, including sensitivity, specificity, and overall accuracy.

### Gold standard for determining diagnostic accuracy

Depending on the clinical indication for FDG PET/CT referral, the reference standard for assessing active myocardial inflammation was based on the 2019 revised JCS guidelines [10]. These guidelines define separate diagnostic algorithms for isolated CS and cardiac involvement in extraCS, incorporating histological and clinical diagnostic groups when biopsy is not feasible or negative despite high clinical suspicion.

In the FU-setting, inflammation was assessed clinically based on symptoms (e.g. dyspnea, chest pain, palpitations), electrocardiographic/echocardiographic findings (e.g. dyskinesia, nsVT or heart failure with reduced ejection fraction [HFrEF]), and laboratory markers (e.g. elevated myoglobin or soluble interleukin-2 receptor [sIL2R]), with active myocardial inflammation defined by at least two criteria from two distinct subgroups. Medication regimens were also considered, with infliximab or cyclophosphamide classified as third-line therapy for treatment resistance. Maintenance therapy included prednisolone (≤ 10 mg/day) or methotrexate (MTX), either as monotherapy or combined with low-dose prednisolone.

### Statistical analysis

Statistical analyses were performed using IBM SPSS Statistics, version 30.0.0 (IBM Corp., Armonk, NY, USA). Clinical and demographic characteristics are presented using descriptive statistics. Data are given with mean and standard deviation or median range depending on distribution.

Intrarater reliability, assessing the agreement between 60-minute and 90-minute acquisitions for each reader, was calculated using Cohen’s kappa (κ). Interrater reliability, evaluating the agreement among different readers per each time points, was determined using Fleiss’ kappa (κ). Kappa values were interpreted according to benchmarks by Landis and Koch [19]: <0.20 (poor), 0.21–0.40 (fair), 0.41–0.60 (moderate), 0.61–0.80 (substantial), and > 0.81 (almost perfect).

Diagnostic accuracy was assessed by calculating sensitivity, specificity, positive predictive value (PPV), and negative predictive value (NPV) using the predefined gold standard. Systematic differences in the majority votes and diagnostic accuracy between the 60-minute and 90-minute uptake time were analyzed using the McNemar-Bowker test. A p-value < 0.05 was considered statistically significant.

**Table 1:** Patient demographics and indications for referred FDG PET/CT

Patient demographics	
Female, N (%)	45 (51.7)
Male, N (%)	42 (48.3)
Age in years, median (range)	55 (25–87)

Indications	
Suspected isolated CS, N (%)	47 (54.0)
Suspected cardiac manifestations with proven extracardial CS, N (%)	20 (23.0)
Proven CS under immunosuppressives treatment, N (%)	20 (23.0)

N: count; CS: cardiac sarcoidosis

## Results

### Patient cohort

In total, 87 FDG PET/CT scans were included in the study. The mean overall blood glucose was 97 (range, 64–199) mg/dl. UFH was administered in 63 patients (72.4%).

Table 2 shows the electrocardiographic and echocardiographic findings observed in patients at the time of initial diagnosis. In patients with confirmed extraCS, diagnostic confirmation was most frequently obtained from pulmonary tissue samples and thoracic lymph node biopsies (Table 3). Patients under FU for potential immunosuppressive dose adjustments underwent FDG PET/CT every six months. The majority of patients received maintenance therapy with prednisone (< 10 mg/day, 60%, 12/20), either alone or in combination with MTX, while 5% (1/20) received MTX monotherapy, and 15% (3/20) had discontinued immunosuppressive treatment. In 20% (4/20) of refractory cases, therapy was escalated, including infliximab (with MTX or prednisone), cyclophosphamide monotherapy, or prednisone with azathioprine.

**Table 2:** Ecg- and echocardiographic findings in patients with suspected isolated cardiac sarcoidosis and suspected cardiac involvement in histologically confirmed extracardiac sarcoidosis

Isolated CS	48 (100)
HFrEF, N (%)	*16 (33.3)*
nsVT, N (%)	*8 (16.7)*
High degree AVB, N (%)	*7 (14.5)*
Regional wall abnormalities, N (%)	*6 (12.5)*
HFmrEF, N (%)	*4 (8.3)*
sVT, N (%)	*3 (6.3)*
Arrhythmia of other origin (AF, SSS), N (%)	*3 (6.3)*
LBBB, N (%)	*1 (2.1)*

ExtraCS	20 (100)
HFrEF, N (%)	*7 (35.0)*
High degree AVB, N (%)	*5 (25.0)*
SSS, N (%)	*2 (10.0)*
LBBB, N (%)	*2 (10.0)*
HFpEF, N (%)	*2 (10.0)*
nsVT, N (%)	*1 (5.0)*
None, N (%)	*1 (5.0)*

HFrEF: heart failure with reduced ejection fraction; nsvt: Non sustaining ventricular tachycardia; AVB: atrioventricular block atrioventricular block; hfmref: heart failure with mildly reduced ejection fraction; sVT: sustaining ventricular tachycardia; AF: atrial fibrillation; SSS: sick sinus syndrome; LBBB: left bundle branch block; HFpEF: heart failure with preserved ejection fraction

**Table 3:** Distribution of histologically confirmed manifestation sites of extracardiac sarcoidosis

Side of involvement	
Pulmonary, N (%)	*9 (45.0)*
Thoracic lymph nodes, N (%)	*8 (40.0)*
Striated muscle tissue, N (%)	*1 (5.0)*
Kidney, N (%)	*1 (5.0)*
Occular/Eye bulb, N (%)	*1 (5.0)*

In 53 patients (60.9%), coronary artery disease was excluded by angiography. Four patients (4.6%) had a history of known coronary artery disease or had previously undergone cardiological interventions, such as stent implantation or coronary artery bypass grafting (CABG). Of the 67 patients undergoing PET for an initial diagnosis, cMRI was available for 57 (85.1%) within the three months preceding the PET/CT-scan. However, cMRI findings were inconclusive for the diagnosis of CS in over 50% of these patients (Table 4). Either no sarcoidosis-specific distribution pattern of late gadolinium enhancement (LGE) was identified, and the differentiation from other storage or infiltrative diseases (such as cardiac amyloidosis or hemochromatosis) or other forms of cardiomyopathy (including dilated cardiomyopathy and non-ischemic cardiomyopathy) proved difficult, or a distinction between fibrotic and inflammatory myocardial tissue within LGE could not be made, thereby precluding the assessment of active inflammation. Among the 67 patients referred for initial evaluation (isolated CS: n, 47; extraCS: n, 20), EMB was performed in 18 cases (26.9%). However, CS was confirmed in only 3 of 67 cases (4.5%).

**Table 4:** Availability of cardiac MRI (cMRI) (B) in patients referred to FDG PET scan for initial diagnosis of CS

	cMRI available, *N*	Inconclusive result, *N*	Conclusive result, *N*	No MRI available, *N*	Total, *N*
Isolated CS	43	32	11	4	47
Cardiac manifestations in extraCS	14	4	10	6	20
Total	57	36	21	10	67

CS: cardiac sarcoidosis; extraCs: extracardiac sarcoidosis

### Diagnostic accuracy

According to the gold standard 15 out of 87 patients had active inflammation isolated in CS (isolated CS *n* = 3; extraCS *n* = 8; FU *n* = 4). Following 60 min of FDG uptake, 25 scans were excluded as drop-outs (leaving 62 scans for further evaluation), and after 90 min, 22 scans were deemed drop-outs (resulting in 65 scans for subsequent analysis).

After excluding dropout cases, 10 patients with positive findings remained at 60 min of FDG uptake. All were correctly identified as true positive. This resulted in a sensitivity of 100%, specificity of 96%, positive predictive value (PPV) of 83%, and negative predictive value (NPV) of 100%.

After 90 min of FDG uptake, excluding dropouts, 12 patients with active inflammation remained, of whom 11 were correctly identified as positive. The sensitivity at this time point was 92%, specificity was 92%, PPV was 73%, and NPV was 98%.

The accuracy in detecting myocardial inflammation using FDG PET/CT was 96.8% (95% CI, 92.5–100%), following a 60-minute FDG uptake period and 92.3% (95% CI, 85.8–98.8%), following a 90-minute FDG uptake period. The absolute numbers and test metrics are summarized in contingency Tables 5 and 6. A detailed summary of the ratings, histopathological results, classification according to the respective gold standard, and whether the diagnosis was correctly made is provided in Supplementary Tables 1–3.

**Table 5:** Contingency table and test metrics for 60 min FDG uptake time

Diagnosis of myocardial inflammation according to JCS criteria [10]	Rating positive, *N*	Rating negative, *N*	Total, *N*
Positive	10	0	10
Negative	2	50	52
Total	12	50	62

Test metrics	Value	95% CI	
Sensitivity	100%	72–100%	
Specificity	96%	87–100%	
PPV	83%	55–98%	
NPV	100%	93–100%	
Accuracy	96.8%	92.5–100.5%	

N: count; JCS: Japanese circulation Society; CI: confidence Intervall; PPV: positive predictive value; NPV: negative predictive value

**Table 6:** Contingency table and test metrics for for 90 min FDG uptake time (B)

Diagnosis of myocardial inflammation according to JCS criteria [10]	Rating positive, *N*	Rating negative, *N*	Total, *N*
Positive	11	1	12
Negative	4	49	53
Total	15	50	65

Test metrics	Value	95% C.I.	
Sensitivity	92%	62–100%	
Specificity	92%	82–98%	
PPV	73%	45–92%	
NPV	98%	89–100%	
Accuracy	92.3%	85.8–98.8.8.8%	

N: count; JCS: Japanese circulation Society; CI: confidence Intervall; PPV: positive predictive value; NPV: negative predictive value

The McNemar-Bowker test showed no significant difference in diagnostic outcomes between 60- and 90-minute FDG uptake times, both for majority vote classifications (*p* = 0.22) and accuracy based on JCS criteria (*p* = 0.22).

### Reliability

#### Intrarater-reliability

The intrarater-reliability analysis, using Cohen’s κ, was performed to quantify the consistency of assessments between the 60- and 90-minute evaluations for each individual reader. Following the randomized evaluation, scans were reassigned per patient, and the corresponding ratings were systematically compared. The ratings of reader 1 demonstrated substantial agreement (κ = 0.703, standard error 0.072), whereas readers 2 and 3 exhibited almost perfect agreement (κ = 0.844, standard error 0.051; κ = 0.899, standard error 0.044).

#### Interrater-reliability

The interrater reliability analysis was conducted to evaluate the degree of variability in the ratings among all three readers at both the 60- and 90-minute uptake intervals. At both time points, a comparable Fleiss’ κ was observed (κ = 0.690, standard error 0.047, 95% CI 0.598–0.781; κ = 0.690, standard error 0.046, 95% CI 0.603–0.783) assuming a substantial agreement between readers.

### Patients with true positive or true negative findings

**Fig. 1:**
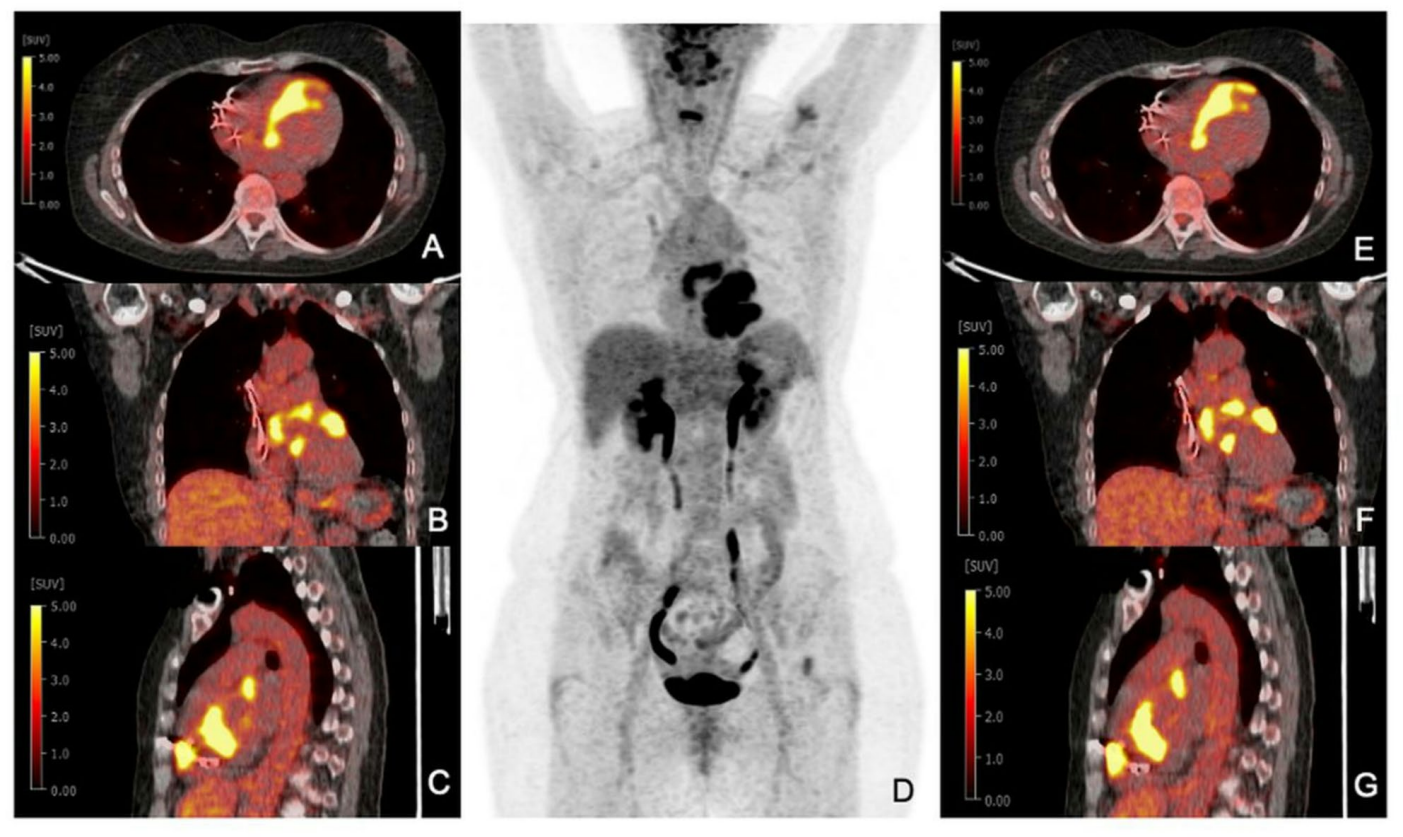
[^18^F]F-FDG PET/CT in patient 28 with suspected isolated CS. **A**-**C** transaxial, coronar and sagittal PET/CT fusion after 60 min of FDG uptake time. **D** maximum intensity projection. **E**-**G** transaxial, coronar and sagittal PET/CT fusion after 90 min of FDG uptake time. Intensive focal FDG uptake in the basal and apical septum with involvement of the right ventricle

A total of 15 out of 87 patients had active inflammation in CS according to the gold standard (isolated CS *n* = 3; extraCS *n* = 8; FU *n* = 4). Among 47 patients referred for suspected isolated CS, diagnosis according to JCS criteria was confirmed in three cases (6%), with EMB available for 14 patients, identifying granulomas in two (patient 5, 28). Both were correctly classified as positive at both time points, showing characteristic patchy FDG uptake, as illustrated in Figure 1.

In the extraCS group, eight patients (40%) were diagnosed with CS, with one confirmed via EMB (patient 52) and seven by clinical criteria based on histologically confirmed extraCS (four from pulmonary biopsies and three from lymph node biopsies via endobronchial ultrasound). In the patient cohort, the following proportions fulfilled the major criteria of cardiac involvement: abnormal high FDG uptake in the heart (100%), left ventricular contractile dysfunction (87.5%), high-degree AVB (62.5%), LGE (50%), and abnormal ventricular wall anatomy (37.5%). All were correctly classified as positive at both time points.

For FU, 20 patients were evaluated, and four were classified as having active myocardial inflammation based on clinical criteria and medication regimen, most commonly presenting with dyspnea (100%), HFrEF (100%), and chest pain (75%). One patient (patient 78) was accurately identified as positive at both time points, while two patient were rated as insufficiently suppressed at both time points (patient 39, patient 67). All patients received third-line immunosuppressive therapy (cyclophosphamide, infliximab, or azathioprine, in some cases in combination with prednisolone or methotrexate). One patient was rated insufficiently suppressed after 60 min and accurately identified as positive after 90 min (patient 79). A summary of patients diagnosed with myocardial inflammation according to the gold standard is presented in Table 7.

**Table 7:** Summary of patients with myocardial inflammation according to the gold standard

	Isolated CS	Cardiac involvement in extraCS	FU	Total
**Patients**,** N**	47	20	20	87
Diagnosis confirmed, total, N	3	8	4	15
EMB available, N	14	4	0	18
Diagnosis confirmed by histology, N	2	1	0	3
Diagnosis confirmed by clinical pathway, N	1	7	4	12

N: count; CS: cardiac sarcoidosis; extracs: extracardiac sarcoidosis; EMB: endomyocardial biopsy; FU: follow up

**Fig. 2:**
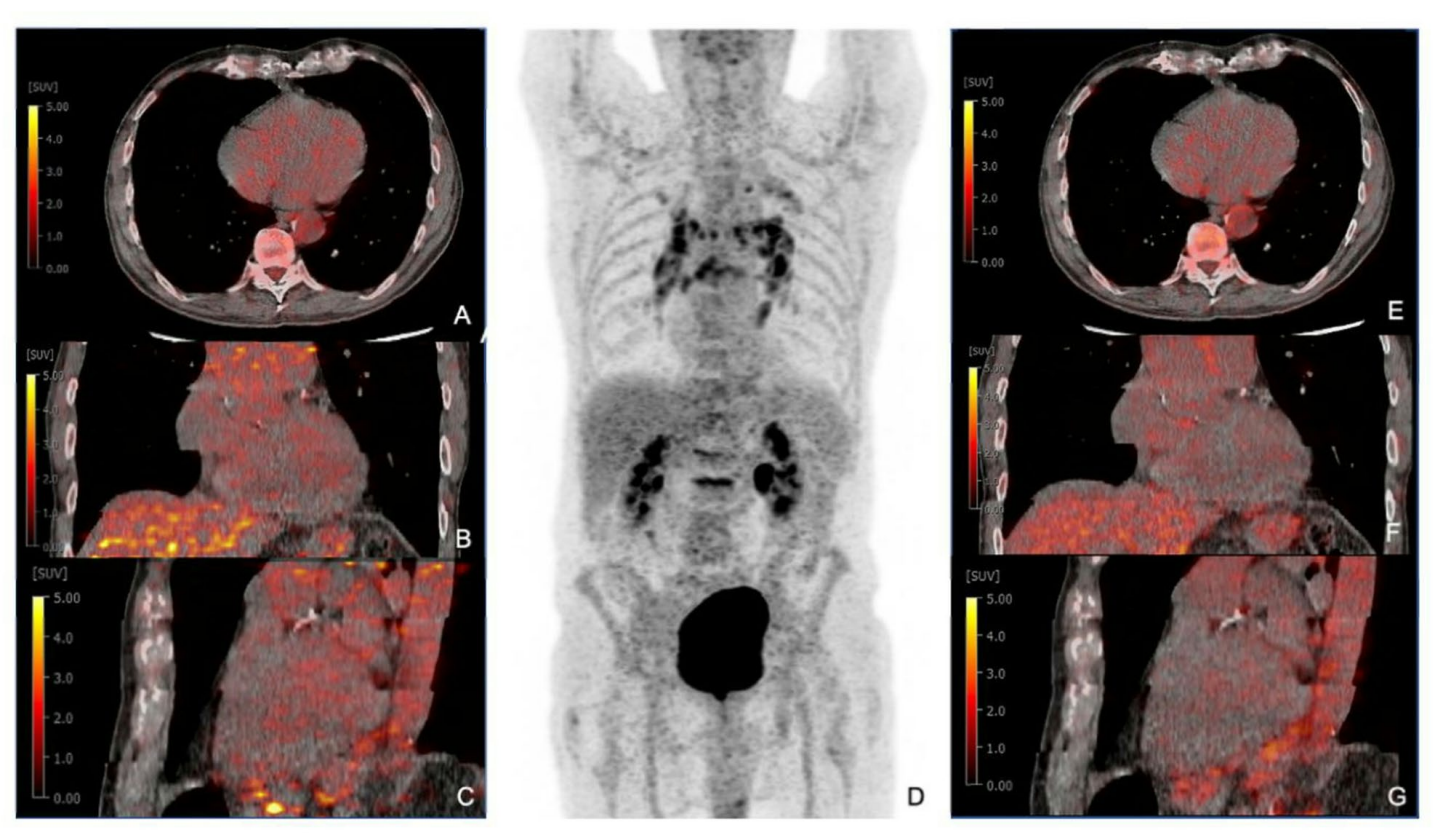
[^18^F]F-FDG PET/CT in patient 71 with suspected cardiac involvement in proven extraCS. **A**-**C** transaxial, coronar and sagittal PET/CT fusion after 60 min of FDG uptake time. **D** maximum intensity projection. **E**-**G** transaxial, coronar and sagittal PET/CT fusion after 90 min of FDG uptake time. No FDG uptake is detected. Extracardiac involvement is shown by hypermetabolism of bihilary lymph nodes

Overall, most patients showed no signs of CS or active myocardial inflammation (isolated CS: 93.6% [44/47], extraCS: 60% [12/20], FU: 80% [16/20]). FDG scans at 60 min post-injection demonstrated higher specificity (96%, 95% CI 87–100%) compared to 90-minute scans (92%, 95% CI 82–98%), though this difference was not statistically significant. Negative scans were defined by the absence of relevant myocardial FDG uptake, with a clearly photopenic septum indicating sufficient suppression. An example is patient 71, with histologically confirmed lymph nodal sarcoidosis but no myocardial involvement, accurately classified as negative at both time points (Figure 2).

### Patients with misclassified diagnosis and differing votes

Among the misclassified cases, four cases were false positive and one case was false negative. The false-positive cases included patients 11, 25, 31, and 68. Patient 11, from the isolated CS group, exhibited a multifocal FDG distribution pattern in both ventricles and met major CS criteria, but EMB confirmed ATTR amyloidosis. Patient 25 showed inhomogeneous and focal apex metabolism and fulfilled major criteria; however, clinical assessment revealed a recent myocardial infarction, attributing the metabolic activity to cardiac remodeling.

Patients 31 and 68 were initially rated as insufficiently suppressed at 60 min and considered dropouts but were classified as positive at 90 min. Patient 31, a FU case with known CS, exhibited faint left ventricular hypermetabolism after having discontinued immunosuppressive therapy for six months. However, in this case, insufficient suppression was assumed. Patient 68, with newly developed HFrEF of 38%, showed diffuse left ventricular metabolism in FDG PET/CT, but cMRI revealed no pathological LGE. EMB ruled out sarcoidosis, confirming non-ischemic dilated cardiomyopathy based on interstitial fibrosis and myocyte hypertrophy.

The only false-negative case was patient 85, diagnosed with CS via the clinical pathway due to unavailable biopsy. The patient exhibited faint FDG uptake in the inferobasal and septal myocardium, initially rated as insufficiently suppressed (60 min p.i.) and negative (90 min p.i.). However, cMRI revealed a clear correlation between FDG uptake and LGE. Given additional clinical criteria, including AVB, dyskinesia, and reduced LVEF, CS was ultimately confirmed.

Diverging findings exclusively occurred when insufficient suppression was rated at either 60–90 min. Notably, no patient was classified as positive at one time point and negative at the other. Notably no differing votes regarding insufficient suppression were found in the extraCS group. Table 8 provides an overview of the misclassified cases and patients with differing votes at 60 min and 90 min uptake duration, their definitive diagnoses, and the diagnostic modalities used for confirmation.

**Table 8:** Patients with misclassified findings or differing votes

Patient	Indication for PET	Majority vote 60 min *p*.i.	Majority vote 90 min *p*.i.	Diagnosis according to JCS-criteria or clinical criteria for FU	True diagnosis	Confirmation of true diagnosis
11	Isolated CS	Positive	Positive	Negative	ATTR-Amyloidosis	Histology
12	Isolated CS	Negative	No suppression	Negative	Lymphocyte myocarditis	Histology
25	Isolated CS	Positive	Positive	Negative	ACS	Clinical symptomts, ecg-, laboratory findings
31	FU	No Suppression	Positive	Negative	Insufficient myocardial suppression	Clinical criteria, medication regiment
68	Isolated CS	No suppression	Positive	Negative	DCM	cMRI, Histology
79	FU	No suppression	Positive	Positive	Active inflammation in isolated CS	Clinical criteria, medication regiment
85	Isolated CS	No suppression	Negative	Positive	Isolated CS	cMRI

JCS: Japanese circulation Society; FU: follow-up; CS: cardiac sarcoidosis. ACS: acute coronar syndrome; DCM: dilatative cardiomyopathy. cMRI: cardiac MRI

## Discussion

This retrospective study assessed the impact of [^18^F]F-FDG uptake duration on PET/CT diagnostic accuracy in suspected CS. Results showed comparable performance for 60- and 90-minute uptake times, with diagnostic accuracies of 96.8% and 92.3%, respectively, though this difference was not statistically significant. Intra- and interrater reliability demonstrated substantial to near-perfect agreement. The McNemar-Bowker test confirmed the lack of significant differences in majority voting (*p* = 0.22) and accuracy (*p* = 0.22), indicating that the extended uptake time did not enhance diagnostic reliability.

### Existing guidelines for [^18^F]F-FDG uptake time and role of delayed imaging

To our knowledge, there are no studies that directly compare the diagnostic accuracy of a 60-minute with a 90-minute uptake time in FDG PET/CT imaging for CS. The joint expert consensus statement from SNMMI and ASNC recommends an FDG uptake time of 60 to 90 min, with a preference for 90 min to enhance the TBR and improve diagnostic accuracy [16]. However, many international guidelines do not specify a defined uptake duration, highlighting a significant lack of standardization in clinical practice. While established procedural statements from SNMMI/ASNC (2017) and EANM/EACVI explicitly mention uptake times of 60 to 90 min more recent guidelines from the ESC [6, 7, 20] and the American Heart Association (AHA) [6], fail to provide clear recommendations regarding the optimal uptake time for CS imaging. Despite the assumption that a prolonged uptake time may improve TBR contrast, there is no universally accepted standard whether 60–90 min provides superior diagnostic accuracy. Aikawa et al. described a single case in which delayed imaging at 120 min revealed an additional FDG-avid septal focus with concurrent blood pool washout [21], however no complementary imaging such as cardiac MRI was performed to confirm whether this represented true inflammation or nonspecific uptake. A larger patient cohort with a more systematic evaluation of delayed uptake times at 60 and 100 min has been presented by Manabe et al. [22]. In that study, delayed scans demonstrated a significantly higher SUVmax of cardiac lesions and a significantly lower SUVmean of the blood pool, leading to a higher lesion-based detection rate of significant myocardial uptake with delayed imaging (100% vs. 91.3%). The reported differences in semiquantitative parameters such as SUVmax and SUVmean did not translate into significant differences in diagnosing cardiac sarcoidosis, underscoring that diagnostic conclusions rely primarily on image interpretation. It has also been suggested that patients with impaired cardiac function, such as those with reduced LVEF, may exhibit delayed blood-pool clearance of FDG, which could necessitate later acquisitions to avoid misinterpretation of residual vascular activity as myocardial uptake [22]. While this subgroup may therefore benefit from delayed imaging, our data did not demonstrate a systematic diagnostic advantage of later time points.

### Diagnostic accuracy and reproducibility

A recent meta-analysis of retrospective studies reports a sensitivity ranging from 84% to 89% and a specificity ranging from 78% to 83% for FDG-PET in diagnosing CS [20, 23]. Our results for sensitivity and specificity may be slightly superior to previous studies due to the use of a fully digital PET/CT system, ensuring improved image quality, resolution and lesion detectability. Digital PET scanners offer higher spatial resolution, enhanced time-of-flight (TOF) capabilities, and improved contrast recovery, leading to better detection of small and diffuse lesions, especially in moving organs like the heart. Studies indicate up to 27% improved lesion detection compared to analog PET, particularly in metabolically active areas [24], which may be particularly relevant in cardiac sarcoidosis.

Intrarater agreement was substantial to almost perfect (κ = 0.703–0.899), highlighting the robustness of repeated readings for monitoring disease progression and guiding therapeutic decisions, particularly in patients requiring long-term follow-up or maintenance therapy.

Interrater agreement was also substantial (Fleiss’ κ = 0.690), confirming the reproducibility of FDG PET/CT across uptake times and its value as a non-invasive tool in both clinical management and multicenter research.

### Challenges in differentiating CS: misclassification and borderline findings

The misclassified cases in our study illustrate the challenges of FDG PET in diagnosing CS, particularly when suppression is incomplete and other cardiomyopathies mimic sarcoid uptake patterns. One patient (11) initially suspected of CS on early and delayed imaging was ultimately diagnosed with ATTR amyloidosis on biopsy, a phenomenon also reported previously, where FDG uptake suggestive of CS was later confirmed histologically as amyloidosis [25]. Another patient’s uptake pattern (25) was eventually attributed to ischemic remodeling after myocardial infarction, underscoring the importance of considering coronary disease in the differential diagnosis. Two patients (31 and 68) were rated as insufficiently suppressed but appeared positiv at 90 min and were later diagnosed with non-sarcoid cardiomyopathies, illustrating the challenge of separating other cardiomyopathies from patterns of incomplete suppression [26]. In summary, these cases clearly demonstrate the very high metabolic sensitivity of FDG imaging, but also its low disease-specific specificity, since FDG PET depicts all myocardial pathologies associated with increased glucose metabolism. The single false-negative case (patient 85) points to the risk of underestimating inflammatory activity in incomplete suppression, as reported in prior studies regarding cardiovascular inflammatory imaging studies in atherosclerosis [27] and emphasizes the value of multimodal imaging. No discrepant readings occurred in patients with extracardiac sarcoidosis, likely reflecting higher pretest probability and additional diagnostic reference. Overall, these findings show that misclassification arises mainly from suppression quality and disease context rather than uptake duration.

### Limitations and future implications for clinical practice

This study is subject to the inherent limitations of retrospective, single-center research, including limited generalizability. Moreover, the relatively low number of confirmed CS cases (15 out of 87 patients) limits statistical power and may reduce the robustness of subgroup analyses. A high number of dropouts due to insufficient myocardial glucose suppression further increases the risk of selection bias. Dropouts were slightly more frequent at 60 min but not statistically significant and did not affect overall diagnostic yield. Variability in dietary adherence and metabolic state likely contributed to suppression failure. However, dietary compliance was not objectively assessed, for example by measuring ketone body levels. Ongoing, unpublished studies at our institution are currently evaluating ketone bodies as potential markers to verify patient adherence and assess scan validity on an individual basis.

A key diagnostic challenge remains the absence of a universally accepted gold standard. Although endomyocardial biopsy (EMB) is considered the reference test, its sensitivity is limited by the patchy distribution of granulomas, often resulting in nonspecific findings such as lymphocytic myocarditis. Even advanced sampling techniques, such as image-guided or voltage map–guided biopsy, offer only marginal improvements in diagnostic yield [28, 29]. Given these limitations, alternative diagnostic frameworks, such as the JCS criteria, are widely used but are still based on expert consensus rather than prospective validation [10]. To improve diagnostic accuracy, standardized multicenter trials are needed to systematically evaluate and refine these criteria.

As this study focused on visual interpretation rather than semiquantitative assessment, ECG-gated imaging was not applied, though future use of gating and motion correction techniques could improve quantitative accuracy and longitudinal monitoring in CS.

Based on the findings of this study, we have adjusted the established clinical standard for imaging in this context by reducing the tracer uptake time from 90 min to 60 min, the standard time interval used in oncology PET imaging as well. A shorter uptake time of 60 min improves patient comfort, enhances workflow efficiency in our departments, and reduces overall scan duration without compromising accuracy. Advances such as whole-body PET scanners and optimized operational protocols further streamline procedures, reducing staff workload, minimizing scheduling delays, and enhancing patient-centered care [30, 31]. Additionally, shorter uptake times improve patient compliance, particularly in elderly individuals and those with limited mobility, while also increasing cost-effectiveness by optimizing PET/CT scanner utilization [31].

## Conclusion

This study demonstrates that a 60-minute FDG uptake time in PET/CT imaging for CS offers high diagnostic accuracy, comparable to a 90-minute uptake time. Importantly, no statistically significant inferiority of the 60-minute time point was observed. The high intra- and interrater agreement in our multi-reader setting further supports the robustness of this protocol. Despite the retrospective nature of this study, the results are highly relevant for clinical adaptation, offering a streamlined approach that benefits both patients and interdisciplinary referring physicians. Given that CS is a rare condition, achieving a high degree of standardization while maintaining maximum diagnostic precision is crucial. The integration of optimized myocardial suppression techniques and multimodal imaging, will further refine diagnostic accuracy, ensuring both harmonization and improved clinical decision-making.

## Supplementary Information

Supplementary Material 1

Supplementary Material 2

Supplementary Material 3

Supplementary Material 4

## Data Availability

The datasets generated during and/or analysed during the current study are available from the corresponding author on reasonable request.
