# Supplementary material for: Optimizing PET/CT protocols: is 60-minute [18 F]F-FDG uptake sufficient for cardiac sarcoidosis?
Source: EJNMMI Res. 2026 Jan 20;16:30. doi: 10.1186/s13550-026-01380-5 (PMC12905051; doi:10.1186/s13550-026-01380-5)
Supplement: Supplementary file 2 — Supplementary Material 2 [file 13550_2026_1380_MOESM2_ESM.docx]

| Patient | majority vote 60 min p.i. | majority vote 90 min p.i. | Histology | JCS-criteria | Diagnosis according to JCS for isolated CS | Correct diagnosis (60 min/90min) |
| --- | --- | --- | --- | --- | --- | --- |
| 5 | Positive | Positive | Cardiac: positive | LGE  LV wall abnormalities  LV contractile dysfunction | Positive | Yes/Yes |
| 6 | No suppression | No suppression | Not available | LV contractile dysfunction  LGE | Negative | -/- |
| 11 | Positive | Positive | Negative (ATTR-amyloidosis) | LGE  LV wall abnormalities  LV contractile dysfunction | Negative | No/No |
| 12 | Negative | No suppression | Not available | LV contractile dysfunktion | Negativ | Yes/- |
| 13 | Negative | Negative | Not available | LGE | Negative | Yes/Yes |
| 14 | Negative | Negative | Not available | AVB | Negative | Yes/Yes |
| 16 | Negative | Negative | Not available | LGE | Negative | Yes/Yes |
| 17 | Negative | Negative | Negative (Parvo-B19) | AVB  LGE | Negative | Yes/Yes |
| 18 | Negative | Negative | Not available | LV contractile dysfunktion  LV wall abnormalities | Negative | Yes/Yes |
| 19 | Negative | Negative | Not available | LV contractile dysfunktion  LV wall abnormalities | Negative | Yes/Yes |
| 21 | Negative | Negative | Negative | AVB  LV contractile dysfunktion | Negative | Yes/Yes |
| 24 | Negative | Negative | Not available | LV contractile dysfunction | Negativ | Yes/Yes |
| 25 | Positive | Positive | Not available | LV wall abnormalities  LV contractile dysfunction | Negative | No/No |
| 28 | Positive | Positive | Positive | LGE  LV contractile dysfunction | Positive | Yes/Yes |
| 33 | No Suppression | No Suppression | Not available | LV contractile dysfunction | Negativ | -/- |
| 34 | No suppression | No suppression | Not available | LGE  LV contractile dysfunction | Negative | -/- |
| 35 | Negative | Negative | Not available | LV contractile dysfunction | Negative | Yes/Yes |
| 36 | Negative | Negative | Not available | LV contractile dysfunction  AVB | Negative | Yes/Yes |
| 40 | No suppression | No suppression | Not available | LGE  AVB  LV contractile dysfunction | Negative | -/- |
| 41 | Negative | Negative | Not available | LGE | Negative | Yes/Yes |
| 42 | No suppression | No suppression | Not available | LV contractile dysfunction  LV wall abnormalities | Negative | -/- |
| 43 | Negative | Negative | Not available | LV wall abnormalities  LGE | Negative | Yes/Yes |
| 46 | Negative | Negative | Negative | LV wall abnormalities  LGE  LV contractile dysfunction | Negative | Yes/Yes |
| 47 | No suppression | No suppression | Negative | LV contractile dysfunction  LGE | Negative | -/- |
| 48 | Negative | Negative | Negative | LV wall abnormalities  LV contractile dysfunction | Negative | Yes/Yes |
| 53 | Negative | Negative | Not available | AVB | Negative | Yes/Yes |
| 54 | Negative | Negative | Not available | LV wall abnormalities  LV contractile dysfunction | Negative | Yes/Yes |
| 55 | Negative | Negative | Not available | LV wall abnormalities  LGE | Negative | Yes/Yes |
| 56 | Negative | Negative | Not available | LV wall abnormalities | Negative | Yes/Yes |
| 59 | Negative | Negative | Not available | LV wall abnormalities | Negative | Yes/Yes |
| 60 | Negative | Negative | Not available | LV wall abnormalities | Negative | Yes/Yes |
| 62 | No Suppression | No Suppression | Negative | LV contractile dysfunction | Negative | -/- |
| 66 | No suppression | No suppression | Not available | - | Negative | -/- |
| 68 | No Suppression | Positive | Negative | - | Negative | -/No |
| 70 | Negative | Negative | Not available | LV contractile dysfunction | Negative | Yes/Yes |
| 73 | No Suppression | No Suppression | Not available | LV contractile dysfunction | Negative | -/- |
| 74 | Negative | Negative | Not available | LV wall abnormalities  LV contractile dysfunction | Negative | Yes/Yes |
| 75 | No suppression | No suppression | Negative | AVB | Negative | -/- |
| 76 | Negative | Negative | Not available | LV wall abnormalities  LV contractile dysfunction | Negative | Yes/Yes |
| 77 | Negative | Negative | Not available | AVB  LV contractile dysfunction | Negative | Yes/Yes |
| 80 | No Suppression | No Suppression | Negative | LGE | Negative | -/- |
| 81 | No Suppression | No Suppression | Negative | LV wall abnormalities  LV contractile dysfunction | Negative | -/- |
| 82 | No suppression | No suppression | Not available | LV wall abnormalities  LV contractile dysfunction | Negative | -/- |
| 83 | No Suppression | No Suppression | Not available | LV wall abnormalities  LV contractile dysfunction | Negative | -/- |
| 84 | Negative | Negative | Negative | LV contractile dysfunction  LGE | Negative | Yes/Yes |
| 85 | No Suppression | Negative | Not available | LV wall abnormalities  LV contractile dysfunction  LGE | Positive | -/No |
| 86 | Negative | Negative | Not available | - | Negative | Yes/Yes |

**SUPPLEMENTARY TABLE 1.** Listing of ratings for patients with suspected isolated CS at 60 and 90 minutes, histopathological results and JCS-criteria. Additionally, it is shown whether the diagnosis was correctly established at the respective time points. CS: cardiac sarcoidosis; JCS_ Japanese Circulation Society; p.i.: post injection; FDG: fluorodeoxyglucose; LGE: late gadolinium enhancement; LV: left ventricle; AVB: atrioventricular block; ACS: acute coronary syndrome
