# Supplementary material for: Optimizing PET/CT protocols: is 60-minute [18 F]F-FDG uptake sufficient for cardiac sarcoidosis?
Source: EJNMMI Res. 2026 Jan 20;16:30. doi: 10.1186/s13550-026-01380-5 (PMC12905051; doi:10.1186/s13550-026-01380-5)
Supplement: Supplementary file 3 — Supplementary Material 3 [file 13550_2026_1380_MOESM3_ESM.docx]

| Patient | majority vote 60 min p.i. | majority vote 90 min p.i. | Histology (cardiac/extracardiac) | Major criteria | Minor criteria | Diagnosis according to JCS with systemic involvement | Correct diagnosis (60 min/90 min) |
| --- | --- | --- | --- | --- | --- | --- | --- |
| 4 | Negative | Negative | Extracardiac: positive (lymph) |  | Abnormal ECG | Negative | Yes/Yes |
| 8 | Negative | Negative | Extracardiac (muscle): positive  Cardiac: negative (ATTR-Amyloidosis) |  | sIL2R+ | Negative | Yes/Yes |
| 10 | Negative | Negative | Extracardiac: positive (opht) | LV wall abnormalities | - | Negative | Yes/Yes |
| 15 | Negative | Negative | Extracardiac: positive (lymph) | AVB | - | Negative | Yes/Yes |
| 20 | Positive | Positive | Cardiac: negative  Extracardiac: positive (lymph) | LGE  FDG  LV contractile dysfunction | - | Positive | Yes/Yes |
| 27 | Positive | Positive | Positive: extracardiac (lymph) | FDG  LGE | Abnormal ECG | Positive | Yes/Yes |
| 29 | Negative | Negative | Extracardiac: positive (lymph) | LV contractile dysfunction | - | Negative | Yes/Yes |
| 30 | Positive | Positive | Positive: extracardiac (pul) | FDG  AVB  LV wall abnormalities | - | Positive | Yes/Yes |
| 37 | Negative | Negative | Positive: extracardiac (lymph) | LGE | - | Negative | Yes/Yes |
| 38 | Negative | Negative | Positiv: extracardiac (lymph, pul) | AVB  LV contractile dysfunction | - | Negative | Yes/Yes |
| 49 | Negative | Negative | Extracardiac: renal  Cardiac: negative | LV wall abnormalities  LV contractile dysfunction | - | Negative | Yes/Yes |
| 50 | Positive | Positive | Extracardiac: positive (lymph) | LV wall abnormalities  LV contractile dysfunction  FDG | abnormal ECG | Positive | Yes/Yes |
| 51 | Negative | Negative | Extracardiac: positive (ren) | LV wall abnormalities  LV | sIL2R+ | Negative | Yes/Yes |
| 52 | Positive | Positive | Extracardiac: positive (pul)  Cardiac: positive | AVB  LV contractile dysfunction  FDG | - | Positive | Yes/Yes |
| 57 | Positive | Positive | Extracardiac: positive (lymph) | AVB  LGE  FDG  LV contractile dysfunction | ECG abnormalities | Positive | Yes/Yes |
| 58 | No suppression | No suppression | Extracardiac: positive (pul) | LV contractile dysfunction  FDG  LV wall abnormalities | - | Positive | -/- |
| 63 | Negative | Negative | Extracardiac: positive (lymph) | LV contractile dysfunction | Abnormal ECG | Negativ | Yes/Yes |
| 65 | Positive | Positive | Extracardiac: positive (pul)  Cardiac: inconclusive | AVB  FDG  LGE  LV contractile dysfunction | sIL2R+  abnormal ECG  LAP | Positive | Yes/Yes |
| 69 | No suppression | No suppression | Extracardiac: positiv (pul)  Cardiac: negative | FDG | LAP | Negative | -/- |
| 71 | Negative | Negative | Extraxardiac: positive (pul, lymph)  Cardiac: negative | AVB | - | Negative | Yes/Yes |

**SUPPLEMENTARY TABLE 2.** Listing of ratings for patients with suspected cardiac manifestations in diagnosed extraCS at 60 and 90 minutes, histopathological results and major/minor JCS-criteria. Additionally, it is shown whether the diagnosis was correctly established at the respective time points. CS: cardiac sarcoidosis; JCS: Japanese Circulation Society; p.i.: post injection; FDG: fluorodeoxyglucose; LGE: late gadolinium enhancement; LV: left ventricle; AVB: atrioventricular block; ECG: electrocardiogram; sIL2R: soluble interleukin-2 receptor; lymph: lymphonodal; LAP: lymphadenopathy; pul: pulmonary; ren: renal; opht: ophthalmic; extraCS: extracardiac sarcoidosis
