# Supplementary material for: Optimizing PET/CT protocols: is 60-minute [18 F]F-FDG uptake sufficient for cardiac sarcoidosis?
Source: EJNMMI Res. 2026 Jan 20;16:30. doi: 10.1186/s13550-026-01380-5 (PMC12905051; doi:10.1186/s13550-026-01380-5)
Supplement: Supplementary file 4 — Supplementary Material 4 [file 13550_2026_1380_MOESM4_ESM.docx]

| Patient | majority vote 60 min p.i. | majority vote 90 min p.i. | Immunosuppressive medication | Active inflammation based goldstandard (symptoms/findings/medication) | Correct diagnosis |
| --- | --- | --- | --- | --- | --- |
| 1 | Negative | Negative | Prednisolon 5mg/d, MTX 15 mg s.c. / week (maintenance) | No | Yes/Yes |
| 2 | No suppression | No suppression | Prednisolon 10mg/d | No | -/- |
| 3 | No suppression | No suppression | MTX 7,5 mg s.c. /week (maintenance) | No | -/- |
| 7 | Negative | Negative | Prednisolon 5mg/d | No | Yes/Yes |
| 9 | Negative | Negative | Prednisolon 5mg/d | No | Yes/Yes |
| 22 | No suppression | No suppression | Prednisolon 1mg/d | No | -/- |
| 23 | Negative | Negative | Prednisolon 10mg/d | No | Yes/Yes |
| 26 | Negative | Negative | Prednisolon 5mg/d | No | Yes/Yes |
| 31 | No suppression | Positive | None (paused) | No | -/No |
| 32 | Negative | Negative | None | No | Yes/Yes |
| 39 | No suppression | No suppression | Infliximab, MTX 7,5 mg s.c./week (maintenance) | Yes | -/- |
| 44 | Negative | Negative | Prednisolon 5mg/d | No | Yes/Yes |
| 45 | Negative | Negative | Prednisolon 5mg/d | No | Yes/Yes |
| 61 | Negative | Negative | Prednisolon 4mg/d | No | Yes/Yes |
| 64 | Negative | Negative | None | No | Yes/Yes |
| 67 | No suppression | No suppression | Cyclophosphamid | Yes | -/- |
| 72 | Negative | Negative | Prednisolon 4mg/d | No | Yes/Yes |
| 78 | Positive | Positive | Prednisolon 10mg/d, Infliximab | Yes | Yes/Yes |
| 79 | No suppression | Positive | Azathrioprin 150mg/d, Prednisolon 5mg/d | Yes | -/Yes |
| 87 | Negative | Negative | Prednisolon 10mg/d | No | Yes/Yes |

**SUPPLEMENTARY TABLE 3.** Listing of ratings for patients in FU examination under immunosuppressive treatment in diagnosed CS at 60 and 90 minutes. Also presented is the medication, the classification as inflammation or non-inflammation based on the gold standard. Additionally, it is shown whether the diagnosis was correctly established at the respective time points. MTX: methotrexate; s.c.: subcutaneous;
