# Supplementary material for: Optimizing PET/CT protocols: is 60-minute [18 F]F-FDG uptake sufficient for cardiac sarcoidosis?
Source: EJNMMI Res. 2026 Jan 20;16:30. doi: 10.1186/s13550-026-01380-5 (PMC12905051; doi:10.1186/s13550-026-01380-5)
Supplement: Supplementary file 1 — Supplementary Material 1 [file 13550_2026_1380_MOESM1_ESM.docx]

Giulia Metzger, M.D. and Christian Furth, M.D.

Charité Centrum 6 – Department of Nuclear Medicine

Charité - Universitätsmedizin Berlin, Campus Virchow-Klinik

Augustenburger Platz 1, 13353 Berlin

GERMANY

Berlin, 15^th^ September 2025

**Subject:** Submission of Manuscript "Optimizing PET/CT Protocols: Is 60-Minute [18F]F-FDG Uptake Sufficient for Cardiac Sarcoidosis?"

Dear Prof. Irene Burger,

We are pleased to submit our manuscript entitled "Optimizing PET/CT Protocols: Is 60-Minute [18F]F-FDG Uptake Sufficient for Cardiac Sarcoidosis?" for consideration for publication in the European Journal of Nuclear Medicine and Molecular Imaging *Research*
We believe that the findings presented in this study are of clinical relevance and contribute to the ongoing optimization of imaging protocols in nuclear cardiology.

Our study demonstrates that a 60-minute FDG uptake time achieves comparable diagnostic accuracy to the currently recommended 90-minute uptake in cardiac sarcoidosis (CS) evaluation. These results not only have direct implications for optimizing patient care but also provide valuable data that could contribute to future guideline development for PET-based diagnostics in CS. Given the increasing demand for PET/CT imaging, the potential to streamline protocols while maintaining diagnostic integrity is highly relevant for clinical nuclear medicine.

We believe that this work will be of interest to your readership, as it addresses an important topic in nuclear cardiology with potential implications for PET/CT standardization. We appreciate your time and consideration of our submission and look forward to your feedback.

Sincerely,

Giulia Metzger, M.D.	Christian Furth, M.D.
